# Cutaneous adverse drug reactions: A prospective observational study at a tertiary care hospital in Central India

**DOI:** 10.6026/973206300215046

**Published:** 2025-12-15

**Authors:** Sudhir Kumar Jain, Pawan Kumar Maurya, Balvir Singh, Sourabh Jain

**Affiliations:** 1 Department of Pharmacology, ABVGMC Vidisha, Madhya Pradesh, India; 2 Department of Pharmacology, Shyam Shah Medical College, Rewa, Madhya Pradesh, India; 3 Department of Pharmacology, Government Medical College, Satna, Madhya Pradesh, India; 4 Department of Dermatology, ABVGMC Vidisha, Madhya Pradesh, India

**Keywords:** Cutaneous adverse drug reactions, fixed drug eruption, pharmacovigilance, NSAIDs, topical steroids

## Abstract

Cutaneous adverse drug reactions (CADRs) are a common and significant concern, arising from both immunological and non-immunological mechanisms. Therefore, it is of interest to identify the incidence, patterns and drug associations of CADRs at a tertiary care hospital in Vidisha, Madhya Pradesh. A total of 56 cases were observed with fixed drug eruptions (FDEs) caused by NSAIDs being the most frequent. The findings highlight the prevalent role of self-medication and the need for strict regulations, with improved pharmacovigilance to mitigate these adverse reactions.

## Background:

Adverse drug reactions (ADRs) are defined by the World Health Organization as "a response to a drug that is noxious and unintended, occurring at doses normally used in humans for prophylaxis, diagnosis, or therapy of disease, or for the modification of physiological function"[1]. ADRs pose a significant burden on healthcare systems worldwide, contributing to increased morbidity, mortality and healthcare costs. Globally, the incidence of ADRs among hospitalized patients ranges from 6% to 10%, [2, 3] with serious reactions contributing to up to 5 % of hospital admissions in some reports [4]. In India, the overall incidence of ADRs has been reported between 2% and 12%, with considerable underreporting due to various systemic and educational gaps [5]. Cutaneous adverse drug reactions (CADRs) are among the most common types of ADRs and often represent the first visible sign of drug intolerance or hypersensitivity. These reactions range from mild manifestations such as urticaria and maculopapular rashes to life-threatening conditions like Stevens - Johnson syndrome (SJS) and toxic epidermal necrolysis (TEN). CADRs account for approximately 10%-30% of all reported ADRs and their identification is crucial for timely intervention and prevention of recurrence [6]. The challenge of managing CADRs is heightened in resource-limited settings, where irrational drug use, self-medication and over-the-counter drug sales are prevalent. Polypharmacy, especially among the elderly and patients with chronic illnesses, further increases the risk of CADRs. Despite the existence of national programs like the Pharmacovigilance Programme of India (PvPI), underreporting remains a critical barrier to effective drug safety monitoring [7]. A study by the Indian Pharmacopoeia Commission (IPC) suggests that less than 5% of actual ADRs are reported, largely due to lack of awareness, time constraints and fear of legal implications among healthcare professionals [8]. In India, the underreporting of ADRs hampers the identification and withdrawal of harmful drugs, emphasizing the need for proactive pharmacovigilance mechanisms. Dermatological ADRs, being readily observable, offer an opportunity for early detection and documentation, provided a robust reporting infrastructure is in place [9]. Therefore, it is of interest to determine the prevalence, patterns and drug associations of cutaneous adverse drug reactions (CADRs), as well as to highlight the role of self-medication and non-prescription drug use, which contribute to these adverse events.

## Methodology:

This was a prospective, observational study conducted jointly by the Departments of Dermatology and Pharmacology at Atal Bihari Vajpayee Government Medical College (ABVGMC), Vidisha, Madhya Pradesh, India. The study lasted for one year, starting from the date of ethical approval by the Institutional Ethics Committee (Approval No.: [02/23/IEC/ABVGMC/Vidisha/2023], dated 04/12/2023). Patients of all ages and both sexes who presented to the dermatology outpatient department (OPD) with suspected cutaneous adverse drug reactions (CADRs) were considered for inclusion. Eligibility was based on a temporal relationship between drug intake and the onset of skin manifestations, including patients presenting with new-onset skin lesions following recent drug use, those willing to give informed consent and both outpatient and inpatient cases reporting dermatological ADRs. Exclusion criteria included patients with incomplete drug histories and reactions caused by food allergens or infections unrelated to drug intake. All patients or their caregivers were interviewed using a structured pro forma. Data collected included patient demographics, drug history (prescription or non-prescription), time of onset of symptoms, description of the reaction and associated systemic symptoms, if any. ADRs were assessed using the WHO-UMC causality assessment scale, which categorizes reactions as certain, probable, possible, unlikely, conditional/unclassified, or unassessable/unclassifiable. Severity of ADRs was classified as mild, moderate or severe based on clinical impact. CADRs were documented through patient self-reporting and confirmed by dermatologists based on clinical examination. In cases where the causative drug was not apparent, detailed medication histories were taken and suspected agents were recorded. Patients were followed up and reactions were recorded until resolution or stabilization. Descriptive statistics were used to summarize demographic details, types of reactions and drug classes implicated. Categorical variables were expressed as percentages and the analysis was performed using Microsoft Excel (Version [Microsoft Excel 2021]. The study was conducted in accordance with the ethical principles outlined in the Declaration of Helsinki and informed consent was obtained from all patients or guardians before inclusion in the study.

## Results:

A total of 56 patients with cutaneous adverse drug reactions (CADRs) were enrolled in the study over a 12-month period. The majority of CADRs were reported in the 31-60 year age group (52.15%), followed by 18-30 years (28.57%) and patients above 60 years (10.71%). Table 1 (see PDF) summarises the age and gender distribution of the study population. Although males presented more frequently to the OPD (ratio 2.3:1), female patients reported a slightly higher incidence of CADRs (54.28%). The most frequently implicated drug class was non- steroidal anti-inflammatory drugs (NSAIDs), accounting for 41.07% of cases and primarily causing fixed drug eruptions (FDE). Topical steroid misuse was the second most frequent cause (12.5%), followed by tinea incognito and irritant contact dermatitis. A summary of CADR types and their frequencies is shown in Table 2 (see PDF). Table 3 (see PDF) presents the severity of CADRs according to clinical judgment. Most reactions were categorized as moderate (87.09%), with a small proportion being mild or severe. Drugs implicated in CADRs were most often acquired without a valid prescription. In 97% of cases, medications were obtained either over-the-counter or through non-qualified practitioners. Only 3% of patients had valid prescriptions. Table 4 (see PDF) highlights the source of the implicated drugs. Finally, Table 5 (see PDF)summarises the causality assessment using the WHO-UMC scale. Most reactions were classified as "probable,"while a few were deemed "possible"or "certain."

## Discussion:

The present study provides real-world evidence on the incidence and patterns of cutaneous adverse drug reactions (CADRs) in a tertiary care setting in central India. Among the 56 cases observed, fixed drug eruptions (FDE) associated with non-steroidal anti-inflammatory drugs (NSAIDs) emerged as the predominant manifestation (41.07%), followed by steroid-induced fungal infections (12.5%) and tinea incognito (7.14%). These findings are consistent with existing literature indicating NSAIDs as a common culprit for CADRs, primarily due to their over-the-counter availability and widespread use without medical supervision. CADRs are a major subset of ADRs because of their visibility and potential to affect patient quality of life, especially when severe forms like Stevens-Johnson syndrome (SJS) or toxic epidermal necrolysis (TEN) occur. Although rare, we encountered one case of SJS, in line with reports from pharmacovigilance studies in India that show SJS and other severe reactions to be less frequent but clinically significant. A significant concern highlighted by this study is the unregulated access to medications: 71.42% of the drugs implicated were obtained directly over-the-counter, while another 25% were prescribed by unqualified practitioners. This lack of regulation creates fertile ground for adverse outcomes, especially with drugs like topical corticosteroids and irrational fixed-dose combinations.

A similar pattern was noted by Makwana et al. (2022) [10], who reported that nearly 40% of CADRs were related to steroid-antifungal-antibacterial combinations used without proper dermatological supervision. Interestingly, our study shares thematic parallels with recent findings on dipeptidyl peptidase-4 inhibitors (DPP-4Is), such as sitagliptin and vildagliptin, which have been associated with hypersensitivity reactions (HSRs), including various cutaneous manifestations. Samajdar et al. (2024) [11] reported drug rashes, urticaria and even SJS linked to DPP-4Is, reinforcing the broad spectrum of cutaneous reactions across therapeutic classes. These reactions, though pharmacologically distinct from NSAID- or steroid-induced CADRs, highlight the immunological complexity and diagnostic challenges posed by drug-induced skin disorders. Another notable observation was the age distribution of CADRs, with over 50% of cases occurring in the 31- 60 year age group. This likely reflects higher drug usage in this demographic due to comorbid conditions and increased exposure to both prescription and non-prescription medications. These trends resonate with findings from broader pharmacovigilance efforts, including those reported by Sharma et al. (2021) [12], which emphasise the vulnerability of the middle-aged population to polypharmacy-related ADRs. Despite the modest sample size, our study provides valuable insights into the real-world landscape of CADRs in India, where patient awareness, pharmacist training and regulatory oversight remain inconsistent. Institutional underreporting to national databases like Vigiflow is another challenge, partly due to lack of Adverse Drug Reaction Monitoring Centers (AMCs) in many hospitals, including ours.

## Conclusion:

Cutaneous adverse drug reactions (CADRs) are a significant concern, particularly due to self-medication and irrational drug use. Thus, we show the need for strict regulations, public education and improved pharmacovigilance. Collaboration among healthcare professionals and the use of digital platforms can enhance ADR detection and minimize preventable reactions.
